# Alternative Splicing Regulation During *C. elegans* Development: Splicing Factors as Regulated Targets

**DOI:** 10.1371/journal.pgen.1000001

**Published:** 2008-02-29

**Authors:** Sergio Barberan-Soler, Alan M. Zahler

**Affiliations:** 1 Department of MCD Biology, University of California Santa Cruz, Santa Cruz, California, United States of America; 2 Center for Molecular Biology of RNA, University of California Santa Cruz, Santa Cruz, California, United States of America; Huntsman Cancer Institute, United States of America

## Abstract

Alternative splicing generates protein diversity and allows for post-transcriptional gene regulation. Estimates suggest that 10% of the genes in *Caenorhabditis elegans* undergo alternative splicing. We constructed a splicing-sensitive microarray to detect alternative splicing for 352 cassette exons and tested for changes in alternative splicing of these genes during development. We found that the microarray data predicted that 62/352 (∼18%) of the alternative splicing events studied show a strong change in the relative levels of the spliced isoforms (>4-fold) during development. Confirmation of the microarray data by RT-PCR was obtained for 70% of randomly selected genes tested. Among the genes with the most developmentally regulated alternatively splicing was the hnRNP F/H splicing factor homolog, W02D3.11 – now named *hrpf-1*. For the cassette exon of *hrpf-1*, the inclusion isoform comprises 65% of *hrpf-1* steady state messages in embryos but only 0.1% in the first larval stage. This dramatic change in the alternative splicing of an alternative splicing factor suggests a complex cascade of splicing regulation during development. We analyzed splicing in embryos from a strain with a mutation in the splicing factor *sym-2*, another hnRNP F/H homolog. We found that approximately half of the genes with large alternative splicing changes between the embryo and L1 stages are regulated by *sym-2* in embryos. An analysis of the role of nonsense-mediated decay in regulating steady-state alternative mRNA isoforms was performed. We found that 8% of the 352 events studied have alternative isoforms whose relative steady-state levels in embryos change more than 4-fold in a nonsense-mediated decay mutant, including *hrpf-1*. Strikingly, 53% of these alternative splicing events that are affected by NMD in our experiment are not obvious substrates for NMD based on the presence of premature termination codons. This suggests that the targeting of splicing factors by NMD may have downstream effects on alternative splicing regulation.

## Author Summary

Alternative splicing is a mechanism for generating more than one messenger RNA from a given gene. The alternative transcripts can encode different proteins that share some regions in common but have modified functions, thus increasing the number of proteins encoded by the genome. Alternative splicing can also lead to the production of mRNA isoforms that are then subject to degradation by the nonsense-mediated decay pathway, thus providing a mechanism to down-regulate gene expression without decreasing transcription. Examples of cell type-specific, hormone-responsive, and developmentally-regulated alternative splicing have been described. We decided to measure the extent of developmentally regulated alternative splicing in the nematode model organism *Caenorhabditis elegans*. We developed a DNA microarray that can measure the alternative splicing of 352 cassette exons simultaneously and used it to probe alternative splicing in RNA extracted from embryos, the four larval stages, and adults. We show that 18% of the alternatively spliced genes tested show >4-fold changes in alternative splicing during development. In addition, we show that one of the most regulated genes is itself a splicing factor, providing support for a model in which a cascade of alternative splicing regulation occurs during development.

## Introduction

Alternative splicing (AS) is the process by which a single pre-mRNA is spliced in different ways to generate multiple mRNA transcripts. This process represents an important mechanism for post-transcriptional regulation of gene expression [1]. It has been estimated that as many as 70% of human genes undergo AS [2],[3]. For *Caenorhabditis elegans*, it has been proposed that ∼10% of genes undergo alternative splicing [4]. Alternative splicing is known to be regulated in tissue-specific, cell-cycle, stress-responsive, hormone-responsive and developmental manners [5]–[7]. The regulation of AS is achieved by an interplay between proteins known as *trans*-acting splicing factors, that can act as activators or repressors, and intronic or exonic RNA sequences known as *cis*-elements, that can also be labeled as enhancers or silencers. With the development of splicing-sensitive microarrays [8] there has been a recent increase in the information about global regulation of splicing in yeast, flies, mice and humans (for a review see [9]).

The establishment of *C. elegans* by Sidney Brenner as a model system for the study of neurobiology and development has led to many important discoveries and advances in biology (for a review of global studies see [10]). Analysis of heterochronic genes led to the discovery of the first microRNAs [11] and studies in this organism led to the discovery of the phenomenon of RNAi [12]. In 1998, *C. elegans* became the first animal to have its genome fully sequenced [13]. The understanding of the complete cell lineage of the animal has led to important insights into development [14]. Even with the wealth of genomic information available about *C. elegans*, there is currently no information about the global regulation of alternative splicing during worm development. Some examples of AS events regulated during *C. elegans* development have been described [15]–[17]. An important question remains as to whether alternative splicing has an important role in the development of this animal. For example, in a global study of alternative splicing it was shown that in Drosophila as much as 46% of the genes show changes in patterns of exon expression during development, suggesting regulated alternative splicing or alternative promoter usage for a high percentage of the genome [18]. In particular, RNAi experiments of several splicing factors showed that some are necessary for worm development while others have redundant functions [19]–[22]. While there is currently no evidence of their targets, it is possible that some splicing factors might function as stage-specific regulators of alternative splicing. This regulation might trigger important changes in diverse cellular processes.

Nonsense-mediated decay (NMD) of messages containing premature termination codons (PTCs) caused by mutation has been described in this organism, and mutations in genes that affect this process have been described as suppressors with morphogenetic effects on genitalia (*smg*) genes [23],[24]. It has been shown that there is a dynamic interplay between alternative splicing and NMD [25]–[27]. While it is known that the inclusion or exclusion of cassette exons changes important functions for diverse proteins, it has also been suggested that the inclusion/skipping events also help the cell to regulate gene expression. Perhaps a gene can be turned off not by turning off transcription but by changing its splicing to produce an isoform of the mRNA that is a target for NMD. This scheme might allow for regulation of individual genes in polycistronic operons in *C. elegans* in which several genes share the same promoter [28]. Thus individual genes in the operon, which could not be regulated individually at the level of transcription, might be regulated at the level of alternative splicing leading to changes in individual mRNA stability. In mammalian cells NMD is known to follow the 55 nucleotides rule, where a PTC needs to be at least 55 bp upstream of the last splice site in order to elicit NMD [29]. It has recently been shown that NMD in *C. elegans* is splicing independent in that it can be elicited by premature termination codons even in intronless genes [30]. This difference in the action of NMD makes the identification of native NMD targets in *C. elegans* more complex.

To add another layer of complexity, the regulation of several splicing factors is known to be achieved by complex interactions that include both alternative splicing and NMD. This was shown originally in *C. elegans* [27] and more recently found in vertebrates [25],[26]. It was demonstrated initially that two *C. elegans* SR protein splicing factors contain exons with premature termination codons (PTCs) that target a particular isoform to NMD [27]. In this regard *C. elegans* presents an interesting model for the study of NMD regulation due to the viability of mutants that are defective in NMD pathways [23]. An important link between NMD and development has been recently shown for *Drosophila*, with the discovery that NMD is highly active during development and is required for the proper expression of dozens of genes as well as for larval viability [31]. However, the link between AS and NMD during development remains to be studied.

In this paper we investigate the regulation of AS during *C. elegans* development, from embryos to gravid adults, and passage through each of the four different larval stages. To do this we developed a splicing-sensitive microarray with features for detection of AS for 352 cassette exons. We show that there is a global regulation of alternative splicing during development, with several events presenting significant differences in AS between different stages. We focus our analysis on events with dramatic changes in AS (greater than four-fold changes in spliced isoform ratios). The highest number of significant splicing changes detected occurred at the step between embryos and the subsequent L1 stage, with 14 AS events highly regulated in this transition. One of the events with the highest change occurs in the W02D3.11 gene, which encodes the *C. elegans* ortholog of the human hnRNP H and F splicing factor proteins, which we have named *hrpf-1*. The skipping isoform of *hrpf-1* includes a PTC that targets a fraction of the messages with this isoform to NMD. However, the exon inclusion isoform shows a dramatic change at the level of splicing at the transition from embryos to L1, with 65% of steady state messages in embryos including this exon but with only 0.1% of messages including this exon in L1s. Global analysis of splicing during development shows the complex nature of this regulation and provides initial clues towards de-convoluting these regulatory networks. We also provide evidence that more than half of the alternatively spliced isoforms of messages that are differentially stabilized in NMD mutant strains appear not to be direct substrates for NMD, suggesting the possibility that NMD regulation of splicing factors may influence downstream alternative splicing events.

## Results

### Microarray Analysis of AS During Development

**Figure 1:**
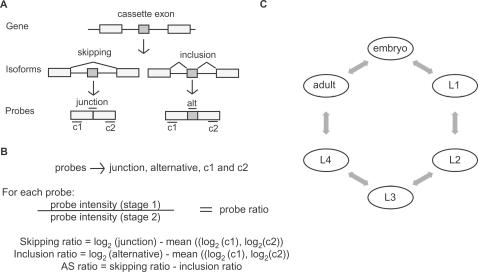
Splicing sensitive microarray design. (A) general outline of the probe design used to detect AS of cassette exons; probes were designed as follows: “alt probe” to detect the cassette exon and thus the inclusion isoform, “junction probe” to detect the splice junction formed in the skipping isoform, and at least two different constitutive probes, c1 and c2, to detect levels of gene expression. (B) AS ratio calculation; (C) loop design to compare all six different time points, each arrow correspond to a direct comparison including the dye-swap experiment (for example, embryo vs. L1 and L1 vs. embryo hybridizations were performed), for a total of 12 microarray hybridization experiments.

We previously reported the identification of a group of 449 *C. elegans* genes with EST or mRNA evidence of having alternative cassette exons [32]. In order to study the regulation of alternative splicing during *C. elegans* development we created a splicing-sensitive microarray with features to detect alternative splicing of 302 of these cassette exons (see Methods and Figure 1 for details). Although the coverage by ESTs in *C. elegans* is limited (∼300,000 ESTs [33]), algorithms to predict alternative splicing events without previous expression evidence have been reported [4],[33],[34]. To further expand the set of AS events detected by our array, we incorporated 50 predictions of cassette exons with AS, based on the RASE algorithm. RASE is a support vector machine-based classifier that identifies known exons with AS potential [34]. In total, our platform has features for the detection of the AS of 352 cassette exons.

**Figure 2:**
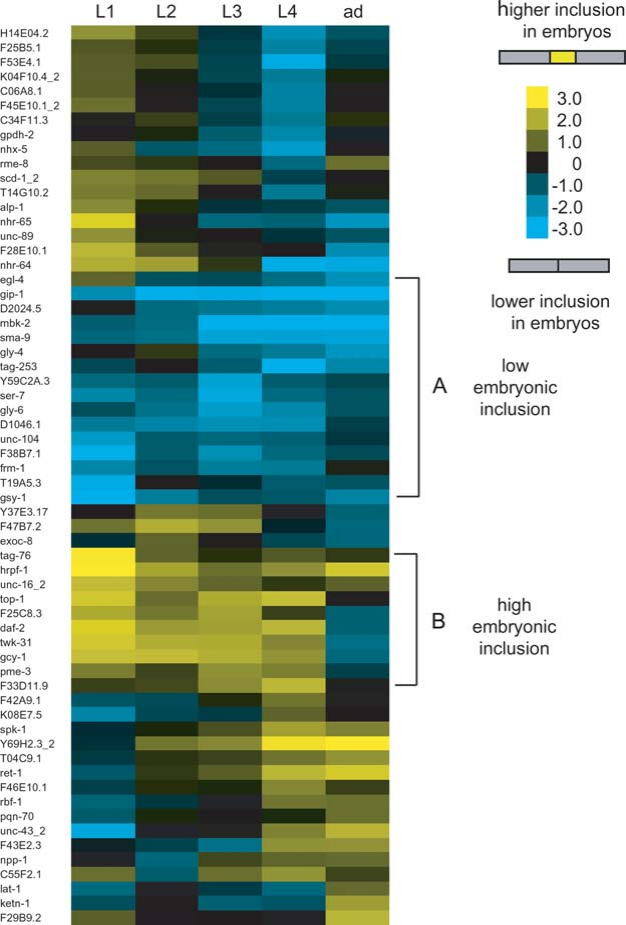
Cluster of top changes. AS ratios showing greater than four-fold differences during development were clustered using hierarchical clustering and TreeView [68],[69]. Positive AS ratios correspond to cassette exons with higher inclusion in embryos, negative AS ratios correspond to higher inclusion in any of the other stages. A and B are two clusters of events with lower and higher inclusion in embryos respectively.

RNA from six different developmental times was obtained from large-scale synchronized cultures; embryo, L1, L2, L3, L4 and gravid adults. To study the changes in alternative splicing during development, we determined the AS ratios for each cassette exon at each developmental stage compared to embryo (set arbitrarily as the reference) (Figure 1B). To do this we calculated the log ratios for each one of the probes (constitutive 1, constitutive 2, alternative and junction) (Figure 1A) between embryo and each of the other five stages. From this embryo vs. stage AS ratio we calculated the AS ratios between all possible pairs of stages to detect all the AS events that changed greater than ±2.0 in log scale. For example: for F25C8.3 the emb vs. L1 AS ratio is 1.94 and the emb vs. adult AS ratio is −1.15 so the L1 vs. adult AS ratio is 3.09. The method for calculation of the AS ratio is shown in Figure 1B. For the microarray analysis we applied a loop design with dye-swaps so that each developmental stage will have four different hybridizations corresponding to two different RNA dye labelings (Figure 1C), comprising a total of 12 hybridizations [35]. We identified 62 cassette exons with AS ratios between any two developmental stages of greater than ±2.0 in log scale (Table 1 and Table S1). These data indicate a greater than 4-fold change in the relative abundance of stable alternatively spliced isoforms containing these cassette exons during the course of development. Clustering of these AS events shows a group of exons that are highly included in embryos (10 exons), another group with low embryonic inclusion (16 exons) and smaller clusters that primarily include the exons at different larval stages (Figure 2). While several of the changes are gradual between subsequent stages, some events present drastic changes between stages (Figure 2).

**Table 1:** Top changes in AS during development.

Gene	Stages with furthest differences	AS ratio (log scale)	RT-PCR confirm	Cassette exon a Multiple of 3?	AS ratio (N2 emb vs. *smg-1* emb)	AS ratio (N2 emb vs. *smg-2* emb)
gip-1	Emb vs. L4	−4.74		no	−0.09	−0.78
mbk-2	Emb vs. Ad	−4.23	-	no	1.17	0.28
gsy-1 (RASE)	Emb vs. L1	−3.75		yes	0.06	1.37
F38B7.1	Emb vs. L1	−3.26	+	no	−0.49	0.78
tag-253	Emb vs. L4	−3.16	+	no	0.48	0.75
nhr-64	Emb vs. L4	−2.84		yes	1.81	−0.45
T19A5.3	Emb vs. L1	−2.76	+	no	0.64	0.93
F53E4.1 (RASE)	Emb vs. L4	−2.74		yes	−0.25	−1.19
K08E7.5	L1 vs. L4	−2.69		no	−0.17	−1.93
ser-7	Emb vs. L3	−2.64		yes	−1.29	−1.19
unc-43_2	Emb vs. L1	−2.63		yes	−2.23	0.21
ketn-1	L1 vs. Ad	−2.59		yes	0.36	0.48
sma-9	Emb vs. Ad	−2.51	+	yes	0.46	0.46
F54A3.3	Emb vs. L3	−2.44		yes	−0.40	−1.1
gly-6	Emb vs. L3	−2.33		yes	−0.92	1.32
nhx-5	Emb vs. L4	−2.32		no	−1.19	1.77
rme-8	L4 vs. Ad	−2.32		yes	−0.53	−0.57
rbf-1 (RASE)	L1 vs. L4	−2.31		no	−1.25	−1.54
unc-104	Emb vs. L1	−2.28	-	no	−0.04	0.49
F43E2.3 (RASE)	L2 vs. Ad	−2.25		no	0.19	−0.16
npp-1	L2 vs. Ad	−2.21		yes	−0.27	−0.17
spk-1	L1 vs. L4	−2.19		no	−0.97	−1.1
gly-4	Emb vs. Ad	−2.18		no	0.24	0.28
lat-1	L1 vs. Ad	−2.16		no	−0.23	0.44
T04C9.1	L1 vs. Ad	−2.12		yes	0.60	0.49
egl-4	Emb vs. Ad	−2.09		yes	−0.14	1.15
pqn-70	L1 vs. Ad	−2.08		no	−1.64	0
H14E04.2	Emb vs. L4	−2.06		yes	2.52	2.24
D1046.1	Emb vs. L3	−2.05		no	1.19	1.75
F42A9.1	L1 vs. L4	−2.04		yes	0.15	−0.49
frm-1	L1 vs. Ad	−2.03		yes	−0.92	−0.9
F46E10.1 (RASE)	L1 vs. L4	−2.03		yes	−1.16	−0.89
scd-1_2	L1 vs. L4	2.00		yes	0.07	1.49
D2024.5	L1 vs. Ad	2.06		yes	1.07	0.4
C55F2.1	L1 vs. L2	2.07		yes	2.02	0.61
C34F11.3	L2 vs. L4	2.09		yes	0.19	0.94
F29B9.2	Emb vs. Ad	2.11	-	yes	1.23	1.46
gpdh-2	L2 vs. L4	2.14		yes	0.19	−0.2
F28E10.1 (RASE)	Emb vs. L1	2.15		no	0.88	−1.06
F33D11.9	Emb vs. L4	2.15	+	yes	−0.68	−1.01
exoc-8	L2 vs. Ad	2.16		no	−0.30	−0.83
pme-3	L3 vs. Ad	2.19		yes	−0.21	−0.75
unc-16_2	Emb vs. L1	2.22	+	yes	−0.26	1.39
gcy-31	Emb vs. L1	2.27		no	−0.98	−1.68
Y37E3.17	L2 vs. Ad	2.33		yes	−0.34	−0.39
alp-1	L1 vs. Ad	2.35		yes	1.37	0.38
F47B7.2	L1 vs. Ad	2.36		no	−0.91	−0.79
twk-31	Emb vs. L1	2.41	-	no	0.73	0.58
top-1	Emb vs. L1	2.42	+	yes	0.29	0.45
K04F10.4_2	L1 vs. L4	2.44		no	2.62	2.15
ret-1_2	Emb vs. Ad	2.45		yes	−0.34	0.79
unc-89	L1 vs. Ad	2.46		yes	−0.03	−0.94
F25B5.1	L1 vs. L4	2.51		yes	−0.87	−0.87
daf-2 (RASE)	Emb vs. L1	2.55		no	0.03	−0.67
nhr-65	Emb vs. L1	2.56	+	yes	1.96	0.67
C06A8.1	L1 vs. L4	2.60		no	1.09	1.36
pxf-1	L1 vs. L4	2.79		no	0.35	1.69
F45E10.1_2	L1 vs. L4	2.83		yes	−0.31	0.23
tag-76	Emb vs. L1	2.91		no	−0.16	−1.42
F25C8.3	L1 vs. Ad	3.09		yes	0.34	−0.68
Y69H2.3_2	Emb vs. Ad	3.17	+	yes	−0.58	−1.17
hrpf-1	Emb vs. L1	4.05	+	no	1.02	−0.52
			Total	58%		

**Figure 3:**
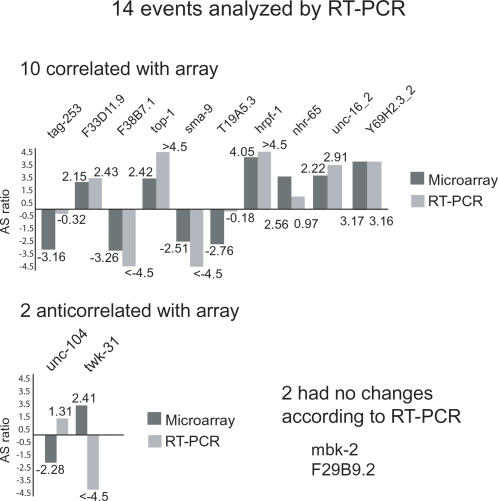
RT-PCR validations of microarray results. From the group of 32 events with AS ratios changes greater than four-fold in development when compared to embryos, 14 events were randomly selected for further confirmation by RT-PCR. AS ratios obtained with the microarray (dark grey), are compared to AS ratios obtained by RT-PCR (light grey).

First column indicates the gene name at Wormbase. The second column shows the developmental stages showing the greatest differences in AS ratio for that gene. “RT-PCR Confirm” indicates those genes whose developmental alternative splicing was tested by RT-PCR, a “+” indicates that there is a correlation between ratios obtained with the microarray and rations obtained by RT-PCR, and “-“ indicates that no changes were detected by RT-PCR or that there is an anticorrelation (for details see Figure 3). Cassette exon a multiple of 3: the size of the cassette exon was measured to determine possible changes in the reading frame by its inclusion/skipping. The last two columns show the AS ratios (log scale) for alternative exon inclusion for N2 embryos vs. *smg-1* mutant embryos or N2 embryos vs. *smg-2* mutant embryos.

In order to confirm these differences in AS we analyzed, by reverse transcription followed by polymerase chain reaction (RT-PCR), the changes in the relative abundance for 14 randomly selected alternative events from the 32 with the highest changes between embryo and any other stage. From this group of randomly selected events we detected AS ratios that correlate with the microarray for 10 events; two did present changes but in disagreement with the microarray, while two showed no changes according to the RT-PCR (Figure 3). This gives an array validation fraction of 10/14 (∼71% with a confidence interval of ±18% for the rest of the AS events with >4-fold changes) for our splicing sensitive microarray. This validation rate is similar to one observed for a comparable microarray platform for detecting AS changes in human breast cancer, in which validation of a subset of array results by RT-PCR confirmed ∼75% of the events predicted by the microarray [36]. Among the cassette exons for which the array data were validated by RT-PCR is topoisomerase-1 (*top-1*), a gene that was previously shown to have an embryo-specific exon [15].

Several of the RT-PCRs show additional bands in addition to the predicted isoforms. For *tag-253* we sequenced one an additional band and identified it as a novel isoform for which there was no previous EST or mRNA evidence. This isoform, which represents ∼65% of the transcripts, derives from skipping of two exons. One of them is the one targeted for detection by the microarray probes, but the other was considered a constitutive exon in the current gene model at Wormbase. This example shows evidence that in some cases, the disagreement between the microarray AS ratios and the ones from the RT-PCR validation can be due to the generation of novel isoforms by alternative splicing that are not considered in the current gene models. Seven of the 62 cassette exons with >4 fold changes in their relative levels during development were novel cassette exons predicted to be alternatively spliced by the RASE algorithm [34] (Table 1 and Figure S1). These changes in levels during development help to confirm that these are indeed alternative cassette exons. In general, these results also validate the RASE approach to alternative splicing prediction; roughly the same fraction of cassette exons showing >4-fold changes in relative usage during development were found in those predicted by RASE (7/50), as for known cassette exons (55/302).

### Alternative Splicing Coupled to NMD

The set of genes showing large changes in alternative splicing during development includes some well characterized genes (i.e. *unc-16*, *unc-43*, *top-1*) and several putative proteins of unknown function (i.e. D1046.1, F28E10.1) (Table 1). Among the characterized genes are two nuclear hormone receptors (*nhr-64* and *nhr-65*). Nhr genes belong to a family of transcription factors known to have an important role in development [37], of which 270 members are found in the *C. elegans* genome [38]. We noted that the alternative spliced exon of *nhr-65* contains a premature termination codon (PTC) that possibly targets this isoform to NMD. Embryos have a higher rate of inclusion for the PTC containing exon of *nhr-65*, opening the possibility that AS coupled to NMD is important for determining the relative steady-state levels of the spliced isoforms of *nhr-65* in embryos.

It has been previously reported that non-symmetrical alternative cassette exons (those not divisible by three) have a higher tendency to disrupt protein domains and to create possible substrates for NMD [39]. Almost half (42%) of the cassette exons with high differences in alternative splicing are not a multiple of three in length. Therefore, inclusion or skipping of these exons by alternative splicing is likely to alter the reading frame for the rest of the transcript, possibly disrupting protein domains and leading to in-frame PTCs (Table 1). While this number (42%) seems high, it is not significantly different (Fisher Exact test, P = 0.42) from the number of exons that are not a multiple of three in the whole set of exons considered for the microarray analysis, where 34% are not a multiple of three (see Table S3).

Given the high proportion of cassette exons undergoing large changes in alternative splicing during development that are not multiple of three, and that some alternative cassette exons contain PTCs, we studied how many of the splicing changes detected are indeed linked to NMD in embryos. To do this, we used the splicing-sensitive microarray to detect the levels of splicing in two strains carrying mutants in NMD genes, *smg-1(r861)* and *smg-2(e2008)*. SMG-1 is a member of the family of phosphatidylinositol 3-kinase (PI3K)-related protein kinases (PIKKs) and is required for NMD [40]. SMG-1 phosphorylates SMG-2/UPF1, the key component of the NMD pathway, and this phosphorylation is required for NMD to occur [41]. RNA was prepared from *smg-1* and *smg-2* mutant embryos and tested on the array in comparison to WT embryo RNA. We found that for 4/62 (∼6%) of the events with high changes during development, the steady state levels of the various isoforms are strongly affected by NMD (greater than 4-fold changes) (Table 1).

In order to see whether there are any specific effects for mutation of either of these two components of the NMD pathway on message stability, we compared the results obtained when comparing WT against *smg-1* or *smg-2* embryo samples (Table 2). 28 different mRNAs showed >4 fold changes in the relative levels of the different isoforms in an NMD mutant background vs. wild type. There are only three genes that show a significant difference in alternative splicing between the two different smg gene mutants. Two of these (akt-1 and C27A7.5) were further analyzed by RT-PCR, and we found no differences in AS according to RT-PCR, so the differences detected by the microarray are false negatives for the *smg-2* sample. This result indicates that the SMG-1 kinase does not have detectable effects on message stability outside of its interaction with its known target, SMG-2.

**Table 2:** Top changes in AS in NMD defective strains.

Gene	N2 vs. *smg-1*(r861)	N2 vs. *smg-2*(e2008)	PTC	Cassette exon a Multiple of 3?
Y55F3AM.3_2	−2.39	−1.81	no	yes
Akt-1	2.2	−0.25	yes	no
Lec-3	−1.35	−2.66	yes	yes
F25B5.7	−2.22	−1.8	yes	no
Unc-43	−2.23	0.21	yes	no
Rsp-6	−2.11	−2.93	yes	no
C05D12.3	2.04	1.86	yes	no
C27A7.5	2.44	−0.03	yes	no
K04F10.4_2	2.62	2.15	yes	no
Swp-1	−2.82	−2.08	yes	no
Y79H2A.3	−2.39	−0.97	yes	no
Y5H2B.2	1.88	3.1	yes	no
Frm-4	−2.35	−2.57	no	yes
C36B1.12	2.16	0.82	no	yes
F36H1.2	2.11	1.74	no	yes
T08G11.1	−2.12	−1.16	no	yes
T01G1.1	2.6	0.73	no	yes
H14E04.2	2.52	2.24	no	yes
F10G8.8	−2.04	−1.34	no	no
C55F2.1	2.02	0.61	no	yes
Klp-4	1.16	2.06	no	yes
D1054.9	0.7	2.03	no	yes
W08A12.1	−0.94	−2.68	no	no
Y97E10AR.2	−2.17	−1.16	no	yes
K04H4.1	1.96	2.06	no	yes
Rig-5	1.85	2.02	no	yes
Ret-1	1.85	2.02	no	yes
Sox-2	−2.18	−1.75	no	yes

The changes in the AS ratios (log scale) for exon inclusion for the indicated genes between N2 embryos and *smg-1* mutant embryos or N2 embryos vs. *smg-2* mutant embryos are indicated. PTC, premature termination codon present in the cassette exon. Cassette exon a multiple of 3: the size of the cassette exon was measured to determine possible changes in the reading frame by its inclusion/skipping.

NMD is known to regulate the mRNA stability of splicing factors isoforms, and in this way creates another level of complexity for determining the relative steady state levels of alternative splicing isoforms in an NMD mutant. This had already been noted for two of the SR protein splicing factors (*rsp-4* and *rsp-6*) [27] one of which did show changes in splicing in a NMD mutant in the present analysis (Table 2). The differences in alternative splicing between wild type and NMD mutant worms may be due to direct effects on the stability of the transcripts (NMD), or to indirect effects due to the mis-regulation of splicing factors.

In general we found that ∼8% of the 352 studied AS events had greater than 4-fold changes in the relative levels of alternatively spliced isoforms in embryos in the *smg-1 or smg-2* mutants when compared to WT (Table 2 and Table S2). If from this 8% we exclude all the events that either contain a PTC (according to the Wormbase gene annotations) or that are not a multiple of three (so they change the reading frame), 53% of the NMD-regulated alternative splicing events are not obvious NMD substrates. This high proportion of cassette exons which are not PTC substrates, but which are affected in an NMD mutant strain, might be secondary targets of the NMD mutations due to the mis-regulation of splicing factors whose mRNAs are direct targets for NMD.

### Alternative Splicing of Splicing Factors

**Figure 4:**
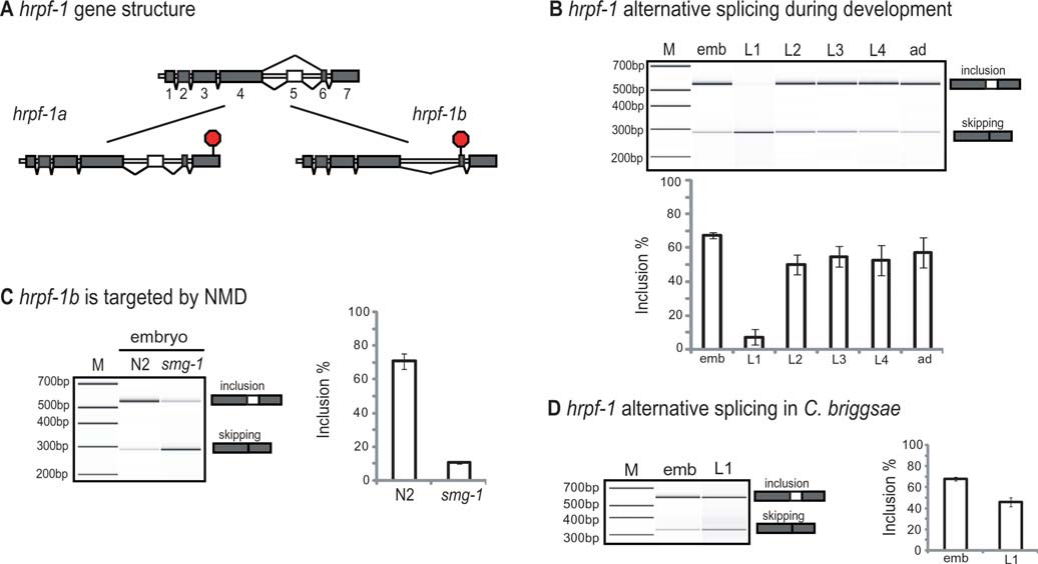
*hrpf-1* alternative splicing. (A) Gene model for AS of *hrpf-1*, two isoforms (a and b) are generated by either inclusion or skipping of exon 5; (B) Virtual gel of RT-PCR of *hrpf-1* during *C. elegans* development as demonstrated on a Bioanalyzer (Agilent). The percentage of *hrpf-1* messages that are spliced to include exon 5 are plotted; (C) RT-PCR of the *hrpf-1* mRNA from total embryonic RNA from NMD defective worms (*smg-1(r861)*) compared to wild type embryo RNA. This experiment demonstrates that *hrpf-1b* is degraded by NMD; (D) regulation of alternative splicing of the *C. briggsae hrpf-1* homolog (CBG04052) during development.

It has been recently shown that the AS of splicing factors is an important step in the global regulation of AS in mammalian cells [25],[26]. Most of the examples reported correspond to the SR family of splicing factors, but there are also some examples of splicing regulation of hnRNPs [42],[43]. In our analysis of AS during development we detected the AS ratio and expression changes for 10 known and putative splicing factors (Table 3). We found differences in the AS ratio of more than 2 log for just one of them, W02D3.11, which encodes the *C. elegans* ortholog of the human hnRNP F protein. We have registered W02D3.11 with Wormbase as *hrpf-1*. The hnRNP F/H proteins are a family of well-studied splicing factors that are known to silence exon inclusion by binding to G rich sequences [44],[45]. Previous work in *C. elegans* has shown that *hrpf-1* together with two other known splicing factors, *unc-75* and *exc-7*, are localized to subnuclear speckles. *hrpf-1* is expressed in all cell types unlike *unc-75 and* exc-7, which are neuronal-specific [46]. Alternative splicing of *hrpf-1* by skipping exon 5 leads to a shorter transcript which still encodes two of the three RRMs but which contains a PTC in exon 6 (Figure 4A). RT-PCR analysis during development shows that *hrpf-1* alternative splicing has a strong regulation in L1 compared to any other stage as suggested by the microarray results (Figure 4B). To further validate the effect of NMD on *hrpf-1* we compared RT-PCR products of N2 and *smg-1* mutant embryonic RNA for this gene. Figure 4C shows that there is a significant difference (∼30% in N2 vs. ∼90% in *smg-1*) in the relative steady state levels of messages showing skipping of exon 5 of *hrpf-1* in embryos of NMD defective worms. This indicates that the skipping isoform is a target for NMD and that alternative splicing skips this exon more often than we can measure in the steady state mRNA level of N2 animals. In addition, since the exon inclusion isoform is not a likely substrate for NMD, its absence in L1 is indicative of a dramatic change in alternative splicing. This strong regulation of a splicing factor allows us to hypothesize that *hrpf-1* may be a regulator of some of the alternative splicing differences that occur in the transition from embryos to subsequent stages. Its own alternative splicing must be tightly regulated in L1, and an understanding of the regulation of the splicing switch of *hrpf-1* will be important to pursue.

**Table 3:** Developmental changes in AS for splicing factors.

Gene	L1	L2	L3	L4	adult	PTC	*smg-1(r861)* embryo
asd-1	−0.13	−0.68	0.36	0.65	0.74	yes	0.71
etr-1	−0.38	−0.72	−0.30	−0.35	−1.29	no	0.10
hrp-1	0.33	0.12	0.12	0.02	−0.18	no	0.98
hrpf-1	4.05	1.86	1.05	1.52	2.41	yes	1.02
ptb-1	−0.67	−0.65	−0.29	−0.05	−0.19	no	0.06
rsp-5	−0.02	0.05	−0.50	−1.04	0.17	yes	−1.57
rsp-6	0.78	0.18	−0.71	−0.05	0.64	yes	−2.11
rsp-7	−0.70	−0.26	−0.75	−0.31	−0.22	yes	−0.38
sup-12	0.49	0.03	−0.24	−0.66	−0.31	no	−1.85
swp-1	−0.54	0.01	0.32	1.37	0.09	yes	−2.82

The AS ratios (log scale) for exon inclusion during development for 10 alternatively spliced, alternative splicing factor RNA binding proteins are indicated. PTC, premature termination codon. The last column indicates the AS ratios (log scale) for N2 embryos vs. *smg-1* mutant embryos for these genes.

In total, our analysis of the NMD defective worms show that at least three of the splicing factors studied have drastic changes in AS, *hrpf-1*, *swp-1* and *rsp-6* (Table 3). Several other splicing factors, including other SR proteins are known to be targeted by NMD [27]. While the differences in AS detected using the microarray in the current work for *hrpf-1* are not as dramatic as the ones found for *swp-1* and *rsp-6,* it does show a difference (1.02 log scale) with more skipping of the cassette exon in the NMD defective worms, in accordance with the semi-quantitative RT-PCR results which show a 3-fold increase in exon skipping (Figure 4C).

To further characterize the developmental regulation of the AS of *hrpf-1*, we asked whether this change is evolutionarily conserved by assaying the levels of splicing for the *C. briggsae* homolog of *C. elegans hrpf-1*. It is estimated that *C. elegans* and *C. briggsae* derived from a common ancestor 100 million years ago [47]. The alternatively spliced cassette exon of *hrpf-1* in the *C. briggsae* homolog has 70% identity with *C. elegans*, and is 3 bp longer than the *C. elegans* exon. As can be seen in Figure 4D, the same type of developmental regulation, where the inclusion isoform is down-regulated in L1s, was detected for *C. briggsae*. The inclusion of the alternative spliced exon goes from 67% in embryos to 48% in L1. While this change is clearly not as dramatic as the one found in *C. elegans*, it does change the major isoform at this developmental stage from exon inclusion to skipping.

### Alternative Splicing Regulation by hnRNP F/H Splicing Factors

**Figure 5:**
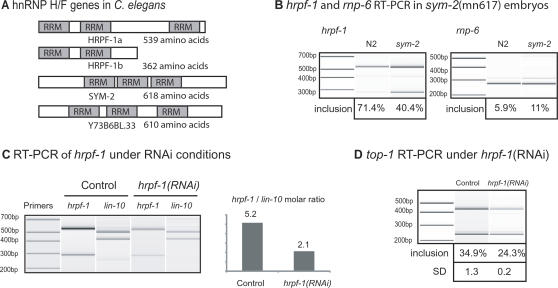
Alternative splicing regulation by hnRNP F/H splicing factors. (A) Graphical representation of the structure of hnRNP H/F proteins in *C. elegans*; (B) RT-PCR of *hrpf-1* and *rnp-6* in N2 and *sym-2*(mn617) embryos. The fraction of mRNA showing inclusion of the alternative exon is indicated below each lane; (C) RT-PCR of *hrpf-1* and *lin-10* under *hrpf-1(RNAi)* and control RNAi conditions. Molar ratios of *hrpf-1* and *lin-10* mRNA levels were calculated using a Bioanalyzer 2100; (D) RT-PCR of *top-1* under *hrpf-1*(RNAi) and control RNAi conditions. The fraction of mRNA showing inclusion of the alternative exon is indicated below each lane.

There are three homologs of the mammalian hnRNP F/H splicing factor proteins in *C. elegans*; *hrpf-1*, *sym-2*, and Y73B6BL.33 (Figure 5A). All three contain three highly conserved RNA recognition motif (RRM) type RNA binding domains. *sym-2* was identified by its synthetic lethality with mutations in another splicing factor, *mec-8* [48]. RNAi of Y73B6Bl.33 results in embryonic lethality, suggesting that this factor regulates the splicing of an essential gene [49].

In order to investigate the role of hnRNP F/H splicing factors in the regulation of alternative splicing, we analyzed global alternative splicing using the splicing-sensitive DNA microarray comparing RNA extracted from wild type and *sym-2(mn617)* mutant embryos. We chose to study splicing in *sym-2* mutant embryos for two reasons. First, the availability of a viable mutant meant that we could obtain the large quantities of synchronized RNA necessary for dye-labeling for the microarrays. Second, because *sym-2* shows higher expression levels in embryos than in L1s [50], alternative splicing changes in the mutant embryos might allow for a correlation with some of the dramatic alternative splicing changes we observed in the embryo to L1 transition in wild type animals. In comparing N2 and *sym-2(mn617)* embryo RNA on the DNA microarray, we observed 65 changes in AS ratios that were greater than 2-fold (Table S3). One striking result was that 6 of the 14 AS events with >4-fold changes in AS during the embryo to L1 transition showed changes in *sym-2* mutant embryos relative to wild type embryos that mimicked the wild type L1 splicing (Table 4). These changes were all in the same direction and strikingly close in magnitude as measured by the array. For example, *twk-31* changes +2.41 (log scale) between embryos and L1 in wt animals and it changes +2.34 between N2 embryos and *sym-2(mn617)* embryos. For these six genes, expression of wild type *sym-2* in embryos appears to account for the splicing difference between embryos and L1. There are an additional seven genes whose alternative splicing both shows dramatic changes during development (but not at the embryo to L1 transition) and whose alternative splicing appears to be regulated by *sym-2* (Table 4). Only two of these seven genes, *ser-7* between embryo and L3 and D2024.5 between L1 and adult, have alternative splicing changes during development that move in the same direction as the *sym-2* mutant embryos compared to wt embryos. Therefore 7 of the 8 genes whose alternative splicing in *sym-2* mutant embryos changes in the same direction as the change during development show one of the two extremes of its developmental splicing regulation in embryos. These data are consistent with *sym-2* having its most important developmental role in embryonic splicing. Among the genes regulated by *sym-2* (Table S3) are two splicing factors, *hrpf-1* and *rnp-6*. *rnp-6* is the *C. elegans* homolog of the drosophila HALF-PINT splicing factor [51]. The changes in alternative splicing for these two genes between N2 embryos and *sym-2(mn617)* embryos were confirmed by semi-quantitative RT-PCR (Figure 5B), consistent with a model in which splicing factors regulate the splicing of additional splicing factors during development.

**Table 4:** Genes regulated by sym-2 that change AS during development.

Gene	Stages with furthest differences	AS ratio (log scale)	N2 Emb vs. *sym-2(mn617)* Emb
F38B7.1	Emb vs. L1	−3.26	−2.03
gsy-1	Emb vs. L1	−3.75	−2.26
hrpf-1	Emb vs. L1	4.05	3.92
T19A5.3	Emb vs. L1	−2.76	−2.32
twk-31	Emb vs. L1	2.41	2.34
unc-43_2	Emb vs. L1	−2.63	−3.59
ser-7	Emb vs. L3	−2.64	−2.84
D2024.5	L1 vs. Ad	2.06	2.49
exoc-8	L2 vs. Ad	2.16	−2.09
rme-8	L4 vs. Ad	−2.32	2.98
ret-1_2	Emb vs. Adult	2.45	−2.24
gly-4	Emb vs. Adult	−2.18	2.11
Y69H2.3_2	Emb vs. Adult	3.17	−2.03

These 13 genes both show >4-fold changes in AS ratios during development and >4-fold changes in N2 embryos vs. *sym-2 embryos. The “stages with the furthest difference” column indicates the developmental stages with the most extreme alternative splicing differences.* AS ratio (log-scale): represents the AS ratio for the stages with the furthest differences. The last column indicates the AS ratios (log-scale) for N2 embryos vs. *sym-2* mutant embryos.

One of the genes with the most similar AS regulation to *hrpf-1* is *top-1*; both show dramatic decreases in exon inclusion during the embryo to L1 transition. According to the microarray analysis of *sym-2* mutant embryos, *top-1* is not strongly regulated by *sym-2*. We decided to ask if *top-1* alternative splicing was potentially regulated by *hrpf-1*. To do this we targeted *hrpf-1* for reduction by RNAi feeding of N2 worms with *E. coli* bacteria expressing double-stranded RNA for a region of *hrpf-1*. We also used an *E. coli* control strain for RNAi feeding that carried only the empty plasmid vector L4440. We isolated RNA from embryos and compared the level of mRNA for *hrpf-1* and a control gene, *lin-10*, between the two differentially fed worm populations. We used the methods established by Caceres and colleagues to estimate the reduction in *hrpf-1* mRNA levels under these RNAi conditions [20]. As shown in Figure 5C, we were able to achieve a modest 60% reduction in *hrpf-1* levels relative to *lin-10* under *hrpf-1* RNAi conditions. We then looked at whether this change led to changes in *top-1* alternative splicing. We consistently detected a 30% relative decrease in *top-1* exon inclusion in *hrpf-1* RNAi embryos relative to a control RNAi feeding (a drop from inclusion in 34.9% of *top-1* messages in the no RNAi control embryos to 24.3% inclusion in *hrpf-1* RNAi embryos) (Figure 5D). Obtaining this modest yet consistent change in alternative splicing with partial removal of *hrpf-1* messages by RNAi is consistent with a model in which *hrpf-1* may play a role in splicing regulation. Changes in *hrpf-1* alternative splicing to an NMD substrate isoform during the embryo to L1 transition may account for some of the alternative splicing changes at this stage of development.

## Discussion

Here we report the first large scale analysis of alternative splicing during *C. elegans* development. Previous studies had demonstrated strong regulation of AS for a small number of individual genes during *C. elegans* development [15]–[17]. In this present work we sought to gain further understanding of the global regulation of AS during development. To achieve this we designed a splicing-sensitive microarray to detect the splicing levels for 352 cassette exons. To validate our splicing-sensitive microarray we used RT-PCR of selected individual genes. With the assumption that the original gene models are accurate, our platform has a validation rate of ∼70% for the detection of changes in AS. This allows us to draw some general conclusions from our microarray studies.

During worm development there are many morphological changes that must occur, including the generation of new tissue types and the increase in size of already existing tissues [14]. A change in the mRNA isoform proportions as detected by the array can be due to a change in the global regulation of AS for a specific gene in many cell types or overrepresentation of particular tissues at specific stages in which that gene undergoes cell type-specific alternative splicing. In both cases, the factors that regulate these splicing events will also have differential representation at the specific stages, even if their expression is limited to a particular cell type.

For some alternative splicing events, a gradual change in steady state levels of AS isoforms is observed between subsequent developmental stages. For example, in *sma-9* the change in emb-L1 AS ratio measured in log scale is −1.24, while the overall change in AS ratio between embryo and adult is −2.51. We found seven AS events in total that presented this type of gradual stepwise change between embryos and adults, and these are reminiscent of the gradual change in mutually exclusive exon usage observed for *let-2* during development [16]. The other major class of alternative splicing changes that we observed showed a dramatic change in the AS ratio between two subsequent stages (i.e. *gip-1*, emb-L1 with a change in AS ratio of −4.74). In this study we found that, for the most part, the major developmental changes in AS ratio in a single developmental step occurred between embryo and L1 (14 events). While we did not address the functional importance of these alternative splicing events, such strong regulation is highly suggestive of a specific function linked to the events. Additional experimentation to show the functions of these highly regulated distinct isoforms will provide further information about specific processes such as transcription or cell signaling (i.e. *nhr-65* and *ser-7*). These changes do not take into account that there are indeed several morphological and molecular changes within the stages used for this study. For example, the development of embryos has several intermediate stages that include the generation of new cell types as well as important molecular events.

To gain further understanding of the regulation of AS during development, and in light of findings that several splicing factors are themselves regulated by AS, we examined the 10 alternative spliced alternative splicing factors for which we had features on the microarray (Table 3). While *swp-1*, *etr-1*, and *rsp-5*, showed greater than 2-fold changes in alternative splicing ratios during development, they paled in comparison to the dramatic 16-fold change in AS ratios observed for *hrpf-1* between the embryo and L1 stages.

*hrpf-1* is a *C. elegans* homolog of the human hnRNP F/H family of splicing factors. In the worm genome there are three genes with strong identity to the hnRNP F/H family: *sym-2*, *hrpf-1* and Y73B6BL.33. We demonstrated that *sym-2* has a role in alternative splicing regulation of many genes in embryos. One striking result was that for 6/14 of the genes showing dramatic changes in alternative splicing between the embryo and L1 stages, the embryo splicing pattern is dependent on *sym-2*. It has previously been shown that hnRNP F/H factors form heterodimers to regulate alternative splicing of a particular target [52], so it is possible that *sym-2* also co-regulates targets with *hrpf-1* or Y73B6BL.33. hnRNP F/H factors in mammals have either activator or repressor activity [52],[53] and they share a similar high-affinity RNA binding sequence [44]. Regulation of splicing by hnRNP F/H family members can also be regulated by competing interactions with SR proteins, whose expression is also regulated [54]. Of the three *C. elegans* hnRNP F/H family members, only *hrpf-1* is known to be alternatively spliced. While it is not known whether the skipping mRNA isoform, *hrpf-1b*, is translated into a protein, the protein it encodes would lack one of the three RNA recognition motifs. This truncated protein could potentially act to interfere with some of the functions of the full-length protein and may lead to an alteration in splicing of target genes. It was recently shown for the human hnRNP F that the C-terminal qRRM is not required for the recognition of RNA G-tracts [44]. This implies that the truncated alternative HRPF-1b protein isoform, if stably produced, may be able to bind specifically to RNA. Future experiments will be required to determine if the dramatic regulation of splicing of *hrpf-1* at the embryo-L1 transition is responsible for regulation of other alternative splicing events in addition to our demonstration by RNAi that *hrpf-1* has a regulatory role in *top-1* splicing. Truncated human SR proteins lacking the C-terminal SR domain have been shown to bind to RNA and alter alternative splicing *in vitro* [55]. The NMD substrate isoforms of *rsp-4* and *rsp-6* similarly encode the RRMs and lack the SR domains. If these truncated isoforms are translated, the proteins produced may have a similar role in regulating alternative splicing. There will be many challenges to work out before a true understanding of the role of hnRNP and SR proteins in splicing can be deconvoluted, but it is clear that both families are important regulators who are themselves highly regulated.

To study the influence of NMD on steady-state mRNA levels for some of these highly regulated AS events, we studied the effects of NMD mutants on AS ratios using the microarray. In *Drosophila*, mutants in the NMD pathway are lethal and it has been suggested that this toxicity in NMD-defective flies might be due to the mis-regulation of native gene expression [31]. While mutants in the NMD pathway are viable in *C. elegans,* our results show that NMD coupled to alternative splicing leads to >4-fold changes in the relative steady-state levels of isoforms of a high percentage of genes (8% of AS events). This result allows us to suggest that the coupling of alternative splicing with NMD is an important step in gene regulation during *C. elegans* development. It is also interesting that several splicing and transcription factors are regulated by alternative splicing and NMD during development and that some of the genes with the most dramatic changes in AS ratios in NMD-defective embryos are themselves not substrates for NMD. This implies that transcripts encoding splicing factors that are targets for NMD (*hrpf-1, swp-1, sup-12, rsp-6*), may be stabilized and translated into proteins with dominant negative effects on splicing if the NMD pathway is defective (Table 3). This may explain why ∼60% of the genes whose AS ratios changed >4-fold in an NMD mutant background are not obvious substrates for NMD regulation and it is also interesting to note that many predicted substrates for NMD regulation do not show dramatic changes in the relative steady-state levels of isoforms in NMD mutant backgrounds. Previous reports have suggested that the activation of NMD in *C. elegans* does not require splicing and it has been suggested that rare codons may help to activate the process [30],[56],[57]. It is clear that more experiments need to be done to understand the rules for activation of NMD in *C. elegans*. Another point to consider is that there are no known phenotypes associated with early development in *C. elegans* NMD mutants. This seems to indicate that this organism in laboratory growth conditions, at least at the early stages of development, can tolerate changes in relative steady state levels of the isoforms of many genes with no detectable phenotypic abnormalities.

**Figure 6:**
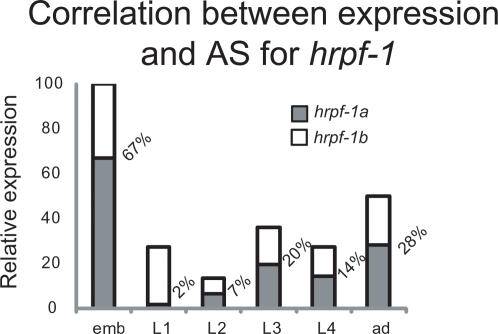
Correlation between steady-state mRNA levels and AS for hrpf-1. Relative levels of *hrpf-1* mRNA during development were derived from published microarray data [58] and alternative splicing levels were determined from our microarray data in Table 1. By regulating the AS of *hrpf-1, C. elegans* is able to further down-regulate the functional transcripts of this gene encoding the full-length, three RRM isoform of this protein in L1 animals.

One potential role of the linkage of AS and NMD in *C. elegans* is to alter the relative expression of genes that belong to the same operon and thus share the same promoter. For example *hrpf-1* is part of an operon that includes two other genes, W02D3.10 and *unc-37*. Results presented here show that in L1s there is a dramatic reduction in the relative levels of *hrpf-1a* isoform to less than 10% of the *hrpf-1* transcripts. Expression microarrays from several groups show that the *hrpf-1*-containing operon has a reduction of around 75% between embryos and L1 [50],[58]. While this reduction affects all three genes in the operon, the regulation of *hrpf-1* is even stronger by the fact that, assuming that *hrpf-1a is* the functional isoform, its expression goes from ∼70% of the transcripts in embryos to ∼10% in L1 (Figure 6). This drastic reduction in the generation of *hrpf-1a* by AS might allow *unc-37* and W02D3.10 to maintain basal levels of expression in L1s, while the levels of functional *hrpf-1* are much more highly reduced.

Developmentally controlled changes in the alternative splicing of alternative splicing factors might initiate a cascade of events that would induce a global effect on gene expression in *C. elegans*. This has been observed in sexual development in drosophila where the sex determination pathway depends on the regulation of alternative splicing of splicing factors at subsequent steps [59]. We demonstrate here that among the genes whose AS events show the most dramatic changes during development there is a splicing factor. We also show that this AS event is coupled to NMD and in that way the strong gene regulation during development is enhanced. Further studies of the effects that *hrpf-1* has on downstream alternative splicing targets (in addition to *top-1*) and identification of the primary and secondary effects of changes in splicing factor regulation will be the next challenges. Future work will be aided by knowledge of the preferred RNA binding sites for the regulated splicing factors on evolutionarily conserved splicing regulatory regions of the primary transcripts.

## Methods

### Alternative Splicing Microarray

BWe previously reported 449 examples of genes with strong cDNA evidence for alternative spliced cassette exons in the *C. elegans* genome [32]. These, together with the 50 predictions made with the Recognition of Alternatively Spliced Exons in *C. elegans* algorithm, were used for the array [34]. For this work we designed microarray probes for all the cassette exons where it was possible to design a junction probe that will be specific for the skip isoforms (i.e. single cassette exons), and where OligoPicker [60] was able to accurately design a probe for that region. We found 352 alternative cassette exons that pass these criteria (Table S1). Our general methodology for probe design can be seen in Figure 1. In brief, we designed 40 bp probes to detect the cassette exon, the junction created by the skipping of the cassette exon, and two constitutive probes for each gene analyzed. The junction probes are centered on the exon-exon junction with 20 bp in each exon. Probes were resuspended in Pronto spotting solution (Corning) at a concentration of 50 µM and robotically spotted onto Epoxide slides (Corning) according to the manufacturer recommendations. Each probe was spotted either four or six times on each slide. The fact that each junction probe has half of its length with perfect complementarity to two different exons that are used in both the skipped and included isoforms creates the possibility of hybridization to both isoforms. This problem has been previously addressed and solved by other array designers. This led us to choose 40 bp oligonucleotides centered on the skipping isoform splice junction as under these hybridization conditions they can discriminate true exon-exon junctions with continuous 40 bp complementarity from skipping isoforms with two 20 bp complementary regions [61],[62]. Probes sequence and raw data are available upon request.

### RNA and DNA Samples

Bristol N2, AF16 (*C. briggsae*), TR1331 (*smg-1(r861)*), CB4043 (smg-2(e2008); him-5(e1490)) and SP2230 (sym-2(mn617)) lines were obtained from the Caenorhabditis Genetics Center. Large quantities of mixed-stage worms were grown on egg-NGM plates with HB101 until plates were confluent; at that point worms were synchronized using 1% sodium hypochlorite and 0.5 M NaOH to isolate embryos. Embryo samples were taken after axenization of adults from mixed-stage cultures. Larval and adult stages were synchronized from embryos that we let hatch overnight in M9 buffer at room temperature as previously reported [63]. The next morning synchronized L1s were washed in fresh M9 and plated onto egg-NGM plates with HB101. Samples were collected at 3 hours (L1), 12 hours (L2), 22 hours (L3), 32 hours (L4) and 48 hours (gravid adults) at 25C. RNA samples were extracted with Trizol reagent according to the manufacturer recommendations (Invitrogen), and further purified using RNeasy columns (Qiagen).

### Hybridizations

20 µg of purified RNA per channel were labeled with Alexa Fluor dyes (555 and 647) using the SuperScript Indirect Labeling System (Invitrogen) according to the manufacturer recommendations for each of the developmental stages of N2 worms as well as for smg-1, smg-2 and sym-2 mutant embryos. Development hybridizations were done in a loop design, with each stage hybridized a total of four times (Figure 1) (i.e. embryo vs. L1, L1 vs. embryo, embryo vs. adult and adult vs. embryo). Hybridizations were done in duplicate with dye swaps. Labeled samples were hybridized to slides for 14–16 hours in 20% formamide, 5× SSC, 0.1% SDS and 0.1 mg/ml sheared salmon sperm DNA. Following hybridization, the slides were washed and dried prior to scanning with an Axon Instruments 4000 series scanners.

### Data Analysis

Data were normalized and further processed using R and Bioconductor [64]. Specifically, Limma “rma” background correction was used to avoid blow out of M-values at low intensities, median normalization was used before differential expression analysis was done with lmFit [65],[66]. Alternative splicing ratios (AS ratios) were calculated as described in Figure 1, [65] and [62]. For comparisons where the RNA samples were not directly compared in the experiments (i.e. embryo vs. L2), the ratio of the probes was calculated by using Limma Contrast Matrix function [65]. Tables containing the AS ratios for all the genes in all experiments are available as Tables S1 and S2.

### RT-PCR

cDNAs from 5 µg RNA samples were synthesized in 20 µl reaction mixtures using oligo dT primers and SuperScript III enzyme according to the manufacturer recommendations (Invitrogen). PCR primers to detect both the inclusion and skipping isoform were designed for the genes indicated in text (primers available on request). 2 µl of the cDNA reaction mixture was used as the template in 25 µl PCR reaction mixtures. Reaction mixtures were incubated between 27–32 cycles at temperatures corresponding to each primer set. PCR products were analyzed using an Agilent Bioanalyzer 2100, with the Agilent DNA 1000 kit. AS ratios and inclusion proportions were calculated from the molar concentrations of each isoform as reported by the Bioanalyzer 2100 software (Agilent).

### RNAi

RNAi by feeding was performed as previously described [67]. A region covering the first 1000 bp of *hrpf-1* was amplified from cDNA (Fwd primer: TCTCGAGGATCAGGCATTCT; rev primer: AGGCCACTGAACAGGAGCTA) and later cloned into the L4440 feeding vector. Plasmids were transformed into bacterial strain HT115. Plates containing 50 µg/ml carbenicillin and 0.1 mM IPTG were seeded with the corresponding transformed HT115 strain (either empty L4440 as control or hrpf-1+L4440). Worms were transferred to IPTG/Carbenicillin plates and let grown for seven days. Collected worms were synchronized as described above. RNAi efficiency was measured by comparing the levels of mRNA for *hrpf-1* and a control gene, *lin-10*, between the two differentially fed worm populations by using semi-quantitative RT-PCR. Molar ratios were calculated using the molar concentration obtained with a Bioanalyzer 2100 as described for the RT-PCR experiments.

## Supporting Information

Figure S1 AS events predicted by RASE in the top 62 changes.
(1.96 MB EPS)

Table S1 AS ratios during development for all 352 events studied.
(0.05 MB XLS)

Table S2 AS ratios between *smg-1*, *smg-2* and WT for all 352 events studied.
(0.06 MB XLS)

Table S3 AS ratios between *sym-2* and WT the top 65 genes.
(0.02 MB XLS)
